# MicroRNA-29 facilitates transplantation of bone marrow-derived mesenchymal stem cells to alleviate pelvic floor dysfunction by repressing elastin

**DOI:** 10.1186/s13287-016-0428-7

**Published:** 2016-11-17

**Authors:** Minfei Jin, Yuelin Wu, Jun Wang, Weiping Ye, Lei Wang, Peipei Yin, Wei Liu, Chenhao Pan, Xiaolin Hua

**Affiliations:** 1 Department of Obstetrics and Gynecology, Xinhua Hospital, Shanghai Jiaotong University School of Medicine, 1665 Kongjiang Road, Shanghai, 200092 China; 2 Department of Obstetrics and Gynecology, Shanghai Sixth People’s Hospital, Shanghai Jiaotong University, 600 Yishan Road, Shanghai, 200233 China; 3 Department of Orthopaedics, Shanghai Sixth People’s Hospital, Shanghai Jiaotong University, 600 Yishan Road, Shanghai, 200233 China

**Keywords:** Pelvic floor dysfunction (PFD), Bone marrow-derived mesenchymal stem cells (BMSC), Elastin, MicroRNA-29a-3p, Nanoparticles

## Abstract

**Background:**

Pelvic floor dysfunction (PFD) is a condition affecting many women worldwide, with symptoms including stress urinary incontinence (SUI) and pelvic organ prolapse (POP). We have previously demonstrated stable elastin-expressing bone marrow-derived mesenchymal stem cells (BMSCs) attenuated PFD in rats, and aim to further study the effect of microRNA-29a-3p regulation on elastin expression and efficacy of BMSC transplantation therapy.

**Methods:**

We inhibited endogenous microRNA-29a-3p in BMSCs and investigated its effect on elastin expression by RT-PCR and Western blot. MicroRNA-29-inhibited BMSCs were then transplanted into PFD rats, accompanied by sustained release of bFGF using formulated bFGF in poly (lactic-co-glycolic acid) (PLGA) nanoparticles (NP), followed by evaluation of urodynamic tests.

**Results:**

MicroRNA-29a-3p inhibition resulted in upregulated expression and secretion of elastin in in vitro culture of BMSCs. After co-injection with PLGA-loaded bFGF NP into the PFD rats in vivo, microRNA-29a-3p-inhibited BMSCs significantly improved the urodynamic test results.

**Conclusions:**

Our multidisciplinary study, combining microRNA biology, genetically engineered BMSCs, and nanoparticle technology, provides an excellent stem cell-based therapy for repairing connective tissues and treating PFD.

## Background

Pelvic floor dysfunction (PFD) is a condition that usually manifests in clinic as pelvic organ prolapse (POP), stress urinary incontinence (SUI), fecal incontinence, and overactive bladder syndrome. Approximately half of parous women suffer from SUI and/or POP to various extents, both of which are closely linked to birth-related injury to the pelvic floor [1]. These disorders occur with increasing rate particularly in adult women of older age [2, 3]. Current clinical treatment and management of these conditions are mainly symptom-based and conservative, and surgeries are only considered for women who fail or decline conservative treatments. Eleven percent of PFD patients above the age of 80 undergo surgery, which often involves partial (anterior or posterior) or total vaginal repair with concurrent hysterectomy [2, 3]. However, as many as 20–30 % of the patients require repeated surgery due to relapse [4, 5]. Recently, the use of synthetic biomeshes during surgical procedures was shown to improve the long-term recovery, but the meshes were also reported to cause pain, erosion, and scarring in about 30 % of cases [6]. Novel treatments are urgently needed to improve the repair and regeneration of damaged tissues in PFD.

Stem cells hold great potential in treating PFD. With their multilineage differentiation ability, they can facilitate tissue repair by potentially differentiating into cells of the connective tissues. Bone marrow-derived mesenchymal cells (BMSCs) are among the best characterized of all stem cells studied so far, with great differentiation ability and being able to secrete factors beneficial for tissue repair [7]. In an animal SUI model, periurethrally injected BMSCs were shown to restore damage to the external urethral sphincter and relieve SUI symptoms [8, 9]. Basic fibroblast growth factor (bFGF) is an FGF family member, and is a growth factor important for the proliferation of mesenchymal stem cells [10, 11], as well as essential to promote the differentiation of the mesenchymal stem cells to fibroblasts [10]. However, bFGF is a short-lived protein, hence to maintain its local concentration stable, multiple and repeated injections are needed, which contributes to the high cost of bFGF-related therapies. In this context, novel and more economic approaches are needed for the maintenance of bFGF local concentration during a reasonable therapeutic period.

Fibroblasts are able to secrete elastin into the connective tissue, and elastic fibers play critical roles in preventing forming or remodeling of collagen into dense tissue. Therefore reconstitution of the connective tissue with the right elastin distribution could be beneficial in alleviating defects in pelvic support. In a recent study, we have demonstrated the efficacy of elastin-expressing BMSC transplant in alleviating PFD symptoms in a rat model [12]. We first genetically engineered BMSCs to stably express and secrete elastin. These elastin-expressing BMSCs were differentiated into fibroblasts under the sustained release of bFGF encapsulated in poly (lactide-coglycolide-co-caprolactone) polymer nanoparticles (bFGF-loaded PLGA NPs). Injection of both the elastin-expressing BMSCs and bFGF-loaded PLGA NPs to the pelvis of PFD rats led to sustained production of collagen and elastin, and promoted tissue repair and regeneration in the PFD rat model.

In the current study, we further expand our work to utilize microRNA-29a-3p (miR-29a-3p), which was known to suppress elastin expression in various earlier reports [13–16]. We first validate that elastin is indeed targeted and repressed by miR-29a-3p in isolated human BMSC culture. Next, by stably inhibiting miR-29a-3p, expression and secretion of elastin are greatly elevated. At last, by employing previously established bFGF-loaded PLGA nanoparticles to allow sustained release of bFGF, we demonstrate that injection of microRNA-29a-3p-inhibited BMSCs into PFD rats in vivo could significantly improve treatment outcome of urodynamic tests.

## Methods

### Isolation and culture of human BMSCs

Tissues were collected from osteotomy sites of patients admitted to Xinhua Hospital, Shanghai Jiaotong University School of Medicine, China. This study involving human tissues was approved by the ethics committee of Xinhua Hospital, Shanghai Jiaotong University School of Medicine, China, and all patients have given written forms of informed consent. All experiments were in compliance with the Helsinki Declaration. Human BMSCs were then isolated by Ficoll centrifugation at 1500 × *g* for 30 min at room temperature. The buffy coat was collected from the interface of Ficoll-HBSS, followed by washing in HBSS. Viable cells were counted with a hemocytometer by trypan blue exclusion, and plated at the density of 50–100 cells/cm^2^ in T75 flasks. Floating cells were removed after 1 day, and the adherent cells were subsequently cultured at 37 °C in 5 % CO_2_ in a humidified incubator.

### BMSC surface marker analysis by flow cytometry

BMSCs were harvested by trypsinization and resuspended in PBS containing 4 % fetal bovine serum. Cells were then stained with FITC-conjugated anti-human CD29, CD90, CD105, and CD45 antibodies (eBioscience, Inc., San Diego, CA, USA). Flow cytometry was performed using FACSDiva (Canto, BD Biosciences, San Jose, CA, USA), and FACS analysis was performed using FlowJo software (Tree Star, Ashland, OR, USA).

### Transfection, microRNA inhibition and assays

The MISSION miR-29a-3p mimic (HMI0434) and miR negative control (HMC0002) were purchased from Sigma-Aldrich (St. Louis, MO, USA), and transfected using Lipofectamine 2000 (Invitrogen, Carlsbad, CA, USA) at 100 nM/1000 cells. Luciferase reporter constructs were transfected using Lipofectamine 2000 (Invitrogen) at 200 ng/1000 cells. The MISSION Lenti hsa-miR-29a-3p Inhibitor Kit (HLTUD0434) and negative control (HLTUD001C) was purchased from Sigma-Aldrich, and was packaged for transduction to create stable cell lines according to the manufacturer’s instructions. MiR-29a-3p expression was determined with TaqMan Advanced miRNA Assay Kit (478587_mir, Applied Biosystems, Waltham, MA, USA) according to the manufacturer’s instructions.

### Reverse transcription polymerase chain reaction (RT-PCR)

Total RNA was purified from cells with the RNeasy Plus Mini Kit (Qiagen, Gathersburg, MD, USA) following the manufacturer’s instructions. The cDNA was synthesized using the QuantiTect Reverse Transcription Kit (Qiagen) following the manufacturer’s instructions. Gene amplification levels were normalized against *GAPDH*. The following primer pairs were used: *elastin* forward 5′-GCC ATT CCT GGT GGA GTT CCT GGA-3′, reverse 5′-ACC GCA CCT GCA GAC ACT CCT AAG-3′; *GAPDH* forward 5′-ACC ACA GTC CAT GCC ATC AC -3′, reverse 5′-TCC ACC ACC CTG TTG CTG T-3′.

### Western blot

Protein samples were extracted using RIPA buffer (50 mM Tris-HCl, 150 mM NaCl, 1 mM EDTA, 1 % Triton X-100, 1 % sodium deoxycholate, 0.1 % SDS, pH 7.4, supplemented with protease inhibitors), and total protein concentration was determined with the Bicinchoninic Acid Protein Assay Kit (Thermo Fisher Scientific, Waltham, MA, US). Proteins were resolved with SDS-PAGE, and subsequently transferred onto nitrocellulose membranes. Primary antibodies against elastin (sc-17581) and actin (sc-8432) were purchased from Santa Cruz Biotechnology (Dallas, TX, USA). Actin was used as loading control. Immunoblot signal was detected using SuperSignal West Pico Substrate (Pierce, Rockford, IL, USA) following the manufacturer’s instructions.

### Enzyme-linked immunosorbent assay (ELISA)

Measurement of human elastin levels in the media was performed by ELISA Kit (Abbexa, Cambridge, UK) according to the manufacturer’s instructions. Briefly, elastin in the media was captured by the specific primary antibody and detected by the biotin-labeled secondary antibody. The assays were developed by avidin-peroxidase and substrate, and absorbance was measured at 450 nm using a microplate reader.

### MTT assay

Cultured BMSCs were plated at 10,000 cells/well in six-well plates. Medium was removed at assay-specific time points, and the wells were washed with PBS twice. A total of 10 μl MTT (0.5 mg/ml) was then added into the wells and incubated for 4 h, followed by absorbance measurement at 570 nm using a microplate reader. Cell viability was determined with the formula: relative viability (%) = (experimental absorbance - background absorbance)/(untreated controls absorbance - background absorbance).

### Rat PFD model

The rat vaginal distention model was established as previously described [12]. Briefly, an 18F catheter was inserted into the rat vagina and then fixed with a single 3-0 silk suture. The Foley balloon was inflated with water (2.5–3.0 ml) and connected to pressure transducer (about 0.15 kg) to create pressure to the pelvic floor support tissue. After 4 h, the catheter was deflated and removed along with the pressure transducer from the vagina. Fourteen days after the vaginal distention, the conscious cystometry (CMG) and leak point pressure (LPP) tests were performed to ensure the successful establishment of the PFD model as well as to evaluate treatment outcomes following injection of BMSCs. This study was carried out in strict accordance with the recommendations in the Guide for the Care and Use of Laboratory Animals of the National Institutes of Health. The protocol was approved by the Committee on the Ethics of Animal Experiments of Xinhua Hospital, Shanghai Jiaotong University School of Medicine.

### Conscious cystometry (CMG) test

Two days before the CMG test, a bladder catheter (PE-50 tubing with a flared tip) was inserted into the PFD rat. The catheter was connected to a syringe pump (KD Scientific, New Hope, PA, USA) and a pressure transducer (Grass Instruments, West Warwick, RI, USA). Each bladder was filled with saline via the catheter at 5 ml/h. A voiding contraction was defined as an increase of bladder pressure that resulted in urine loss as detected by a force transducer (Grass Instruments) that was calibrated to measure volume. Three fills and voids were recorded on each rat. Mean bladder baseline pressure, mean voided volume, mean peak voiding pressure, and mean increase in bladder pressure for voiding (peak voiding pressure minus bladder baseline pressure) were calculated for each animal with a chart recorder.

### Leak point pressure (LPP) test

Two days before the LPP test, a bladder catheter (PE-50 tubing with a flared tip) was inserted into the PFD rat and connected via a stopcock to the pressure transducer and flow pump. Under anesthesia with urethane (1.2 g/kg body weight intraperitoneally), the bladder was palpated to empty and filled with saline at 5 ml/h via the flow pump. When 0.3 ml was attained (approximately half the capacity of a 200 g rat), gentle pressure was applied with one finger to the rat’s abdomen to increase bladder pressure while bladder pressure was recorded and digitized. Pressure was slowly increased until the rat leaked saline through the urethra. At the first indication of leakage at the urethral meatus, the externally applied abdominal pressure was rapidly removed. Peak pressure at leakage in the absence of a detrusor contraction was recorded. LPP was calculated by subtracting bladder baseline pressure from peak bladder pressure. The bladder was drained and refilled, and the study was repeated three times in each rat. Mean bladder baseline pressure and mean LPP were calculated for each rat.

### Statistical analysis

Statistical analysis was performed with the SPSS software (SPSS Inc., version 19.0, Chicago, IL, USA). Values were mean ± SD from at least three independent experiments for all in vitro assays, and from 12 rats in each group for all animal experiments. A two-group comparison was conducted using the unpaired Student’s *t* test, and *P* < 0.05 indicated statistically significant differences.

## Results

### MiR-29a-3p targets elastin in BMSCs

**Fig. 1:**
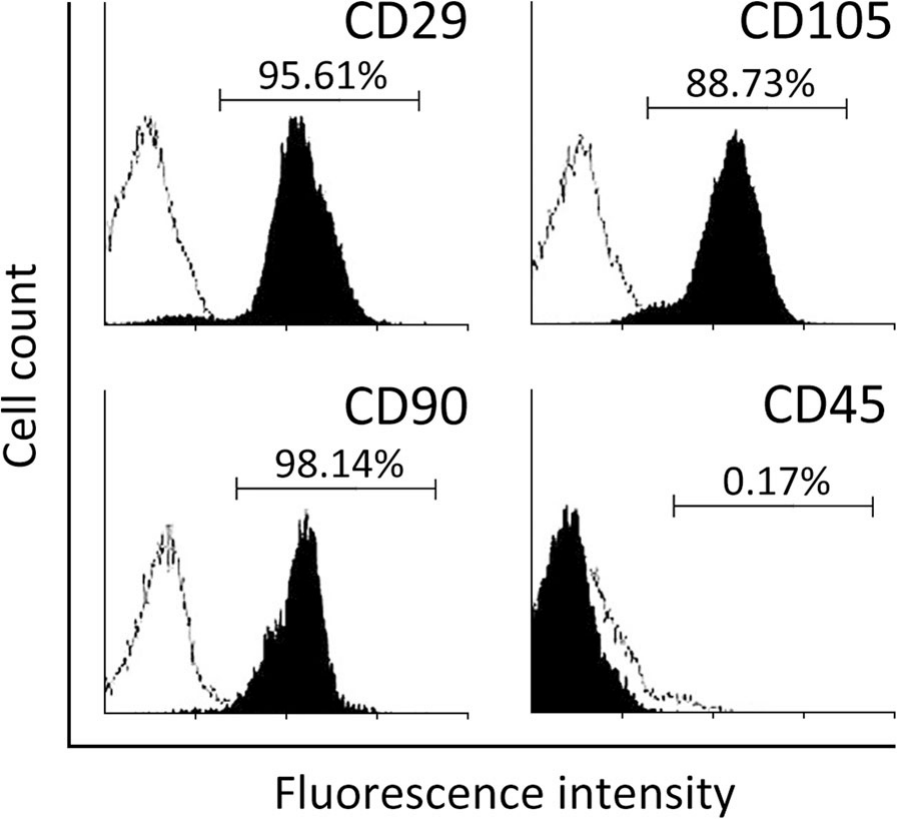
Flow cytometry identifies human BMSC-positive markers CD29, CD90, and CD105, but not negative marker CD45, on surface of the cells, against isotype antibody as control (shown *in white*)

In this study, we first isolated human BMSCs and cultured them in vitro. The identity of these isolated cells was subjected to flow cytometry analysis of the cell surface markers specific for mesenchymal stem cells. As shown in Fig. 1, the isolated cells were positive for CD29, CD90, and CD105, while negative for CD45, which was consistent with reported surface markers of the mesenchymal stem cell lineage [17], thereby confirming the identity of cells used in this current study to be BMSCs.

**Fig. 2:**
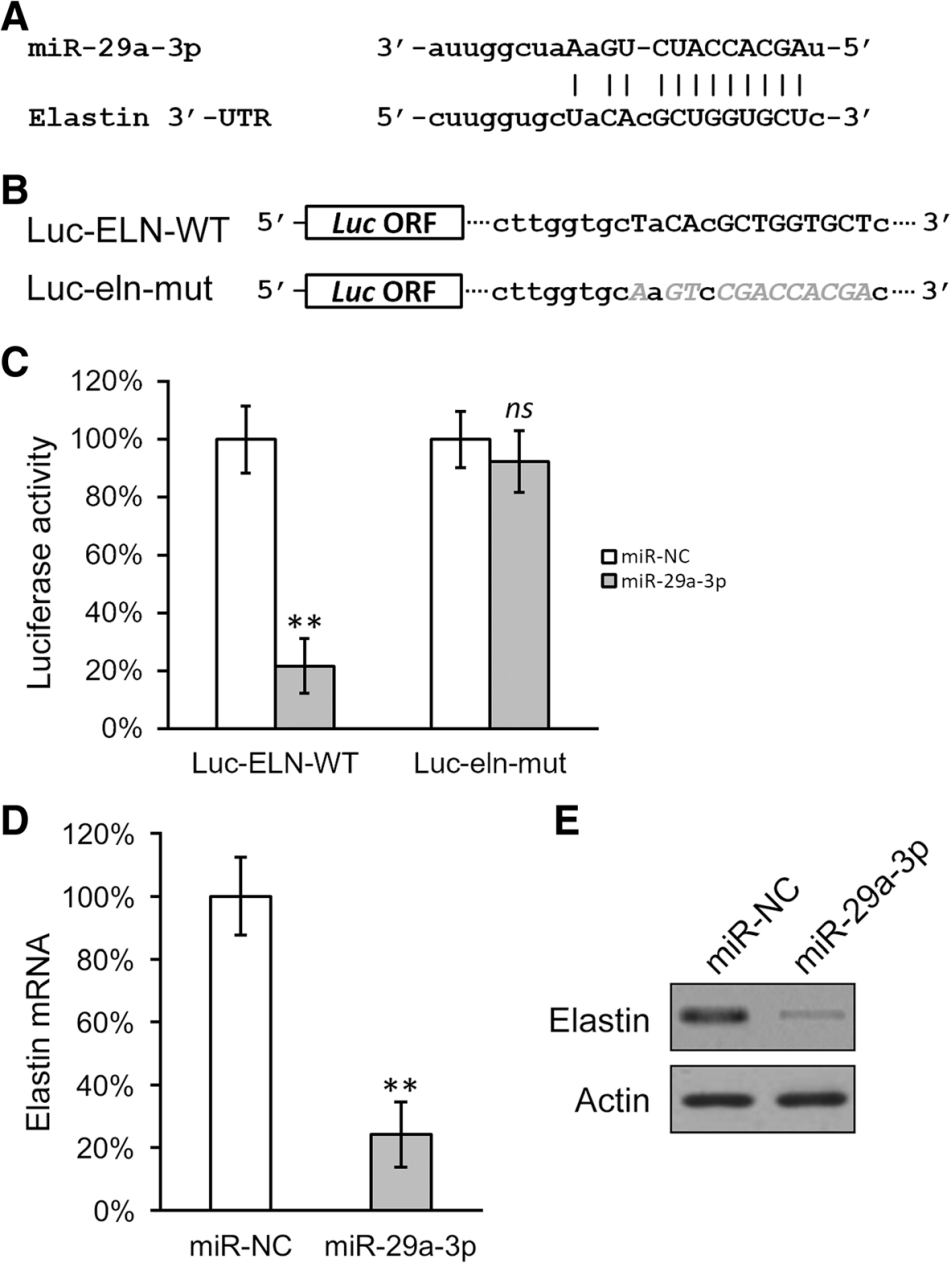
miR-29a-3p directly targets 3′-UTR of elastin mRNA. **a** Sequence alignment of miR-29a-3p with potential targeting site in the 3′-UTR of elastin mRNA. **b** Wild-type (-Luc-ELN-WT) or mutated (Luc-eln-mut) targeting sequences from elastin mRNA 3′-UTR were fused at the 3′ of the luciferase reporter open reading frame (Luc ORF). **c** Luciferase activities of Luc-ELN-WT and Luc-eln-mut constructs were measured in BMSCs transfected with negative control miR (miR-NC) or miR-29a-3p. **d** and **e** Expressions levels of elastin mRNA (**d**) and protein (**e**) were examined in BMSCs transfected with miR-NC or miR-29a-3p. Values were mean ± SD from three independent experiments. ^**^ *P* < 0.01, ns not significant vs miR-NC

We continued to verify whether miR-29a-3p was able to directly target elastin in the isolated BMSCs. Using TargetScanHuman Release 7.1 online resource [18], we found a potential site in the three prime untranslated region (3′-UTR) of elastin mRNA matching the miR-29a-3p sequence (Fig. 2a). This potential binding site on the 3′-UTR of elastin mRNA, as well as its mutated form, was subsequently cloned to the downstream of a luciferase open reading frame (Fig. 2b). Twenty-four hours after these constructs were transfected into cells, together with miR-29a-3p, the activity of the luciferase fused with wild-type targeting sequence was significantly reduced, whereas the mutated version was practically unaffected (Fig. 2c), indicating that this site on the 3′-UTR of elastin mRNA was directly targeted by miR-29a-3p. In fact, transfection of miR-29a-3p into the BMSCs, after 24 hours, greatly repressed both the mRNA and protein expressions of endogenous elastin (Fig. 2d and e).

**Fig. 3:**
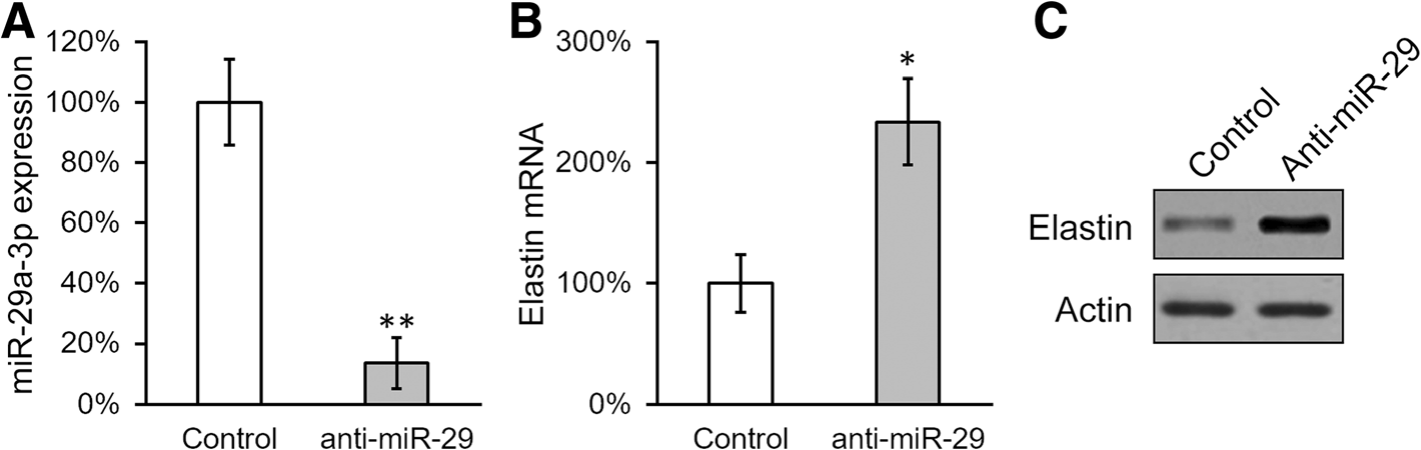
Constitutive inhibition of miR-29a-3p in BMSCs increased steady state expression level of elastin. Expressions of miR-29a-3p (**a**), elastin mRNA (**b**), and elastin protein (**c**) were examined in BMSCs transduced with negative control inhibitor (control) or miR-29a-3p-specific inhibitor. Values were mean ± SD from three independent experiments. ^*^ *P* < 0.05, ^**^ *P* < 0.01, vs control

The above results suggested that miR-29a-3p was a bona fide negative regulator of elastin in BMSCs. If this indeed is the case, depletion of miR-29a-3p in BMSCs should result in an upregulation of elastin. To test this hypothesis, inhibitor of miR-29a-3p was stably introduced in the cultured BMSCs (Fig. 3a). As expected, both the mRNA and protein expressions of elastin were significantly elevated in miR-29a-3p-inhibited BMSCs compared to the control culture (Fig. 3b and c).

### bFGF-loaded PLGA NP promoted the proliferation of miR-29a-3p-inhibited BMSCs

**Fig. 4:**
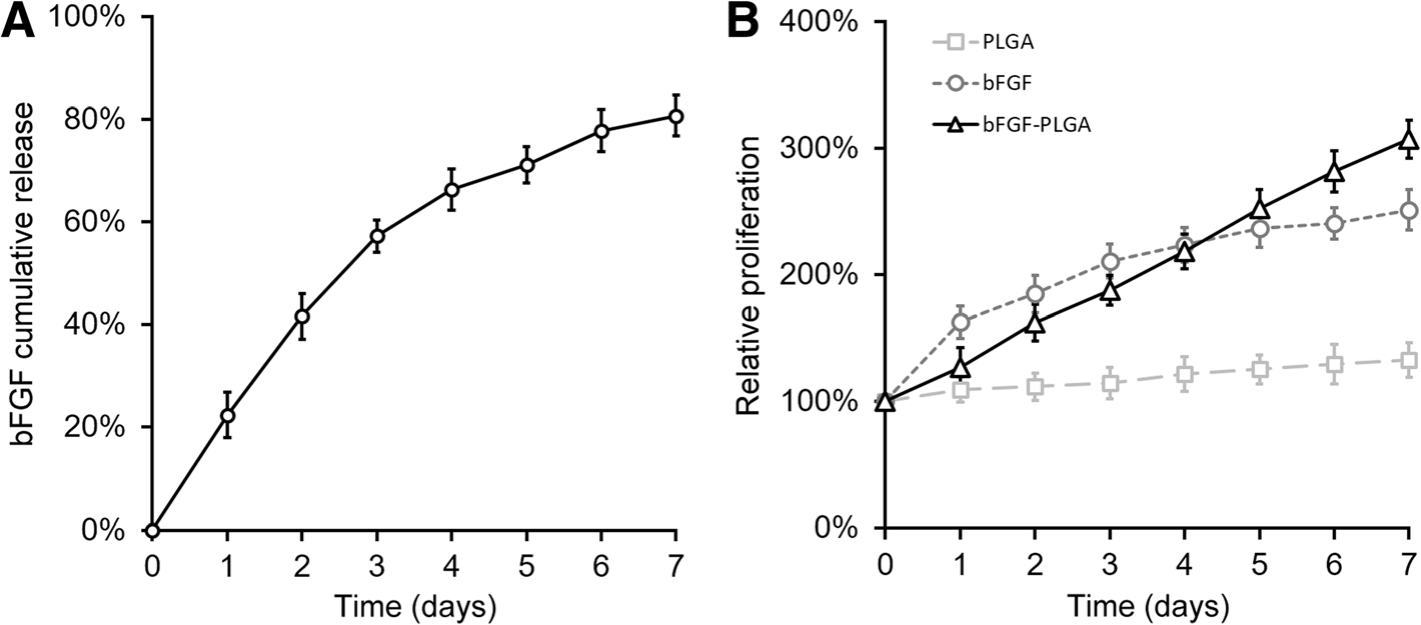
Sustained release of bFGF promoted the proliferation of BMSCs. **a** Cumulative release of bFGF from bFGF-loaded PLGA was profiled in vitro. **b** Cell proliferation was measured by MTT assay, after empty PLGA, free bFGF without PLGA (bFGF), or bFGF-loaded PLGA (bFGF-PLGA) were added into the culture of miR-29a-3p-inhibited BMSCs. Values were mean ± SD from three independent experiments

In our previous work [12], we have demonstrated the effectiveness of the bFGF-loaded PLGA NPs in sustaining continuous proliferation of BMSCs in prolonged culture as the bFGF source. Similarly, we verified the in vitro curve of bFGF release, by incubating the bFGF-loaded PLGA NPs in the culture media for 7 days (Fig. 4a). In line with our earlier results, about 80 % of the initially loaded bFGF was released into the media during the 7-day experiment, at a sustained and steady rate.

We next evaluated the effect of the released bFGF on proliferation of the miR-29a-3p-inhibited BMSCs (Fig. 4b). The cells were cultured in the presence of empty PGLA as negative control (Fig. 4b, PLGA), 20 ng/ml free bFGF (Fig. 4b, bFGF), and 20 ng/ml equivalent bFGF-loaded PLGA NPs (Fig. 4b, bFGF-PLGA), respectively. During the 7-day incubation, the culture was not replenished with any additional bFGF, and proliferation of cells were measured by MTT assay. We found that BMSCs barely grew without bFGF in the empty PLGA control group, and cells with free bFGF exhibited an initial fast-paced proliferation during the first 4 days but slowed down gradually without replenishing bFGF. In contrast, BMSCs incubated with bFGF-loaded PLGA exhibited a relatively slow but steady pattern of proliferation throughout the 1-week-long experiment, albeit slower than free bFGF-treated cells in the first 4 days. Noteworthy, the bFGF sustained release from the PLGA NPs was able to support the continuous proliferation of cells even for prolonged incubation beyond day 5. Therefore consistent with our previous work, bFGF-loaded PLGA NPs sustained release system was also effective for our genetically engineered miR-29a-3p-inhibited BMSCs.

### miR-29a-3p inhibition synergized with bFGF-PLGA in promoting the expression and secretion of elastin in differentiated BMSCs

**Fig. 5:**
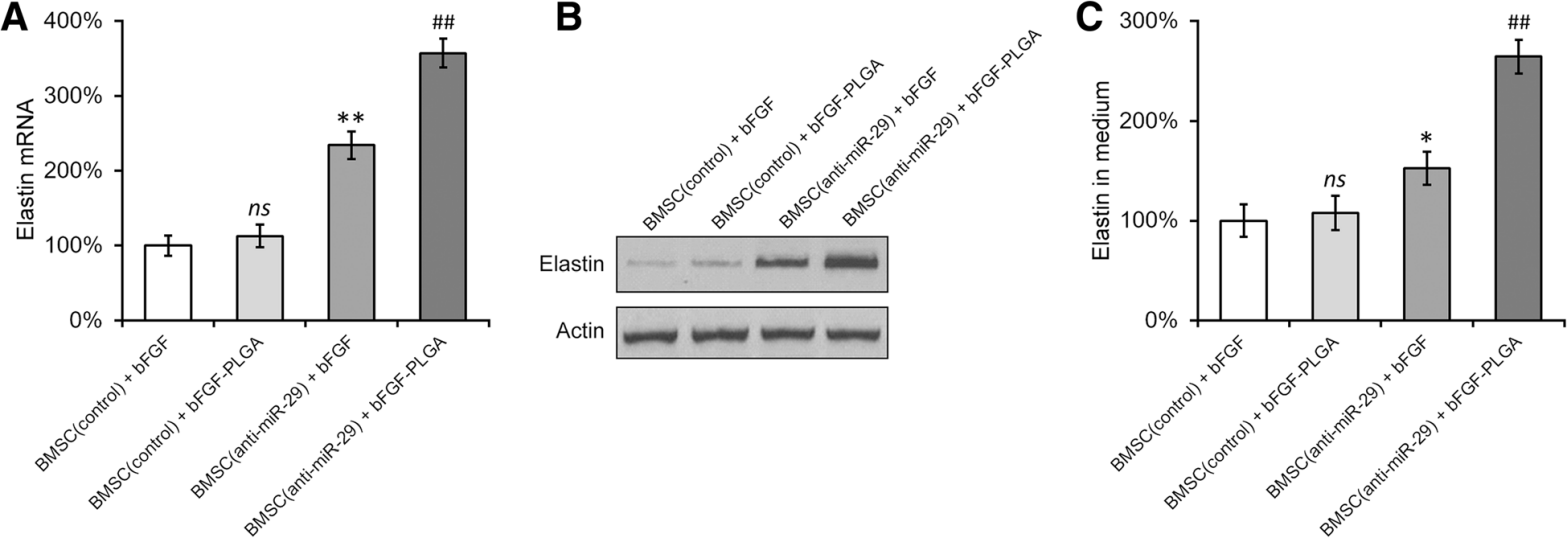
miR-29a-3p inhibition synergized with bFGF-PLGA in promoting the expression and secretion of elastin in differentiated BMSCs. BMSCs transduced with negative control inhibitor (control) or miR-29a-3p-specific inhibitor were differentiated in the presence of either bFGF or bFGF-PLGA for 7 days. Levels of elastin mRNA (**a**), protein (**b**), and secreted elastin in medium (**c**) were examined by RT-PCR, Western blot, and ELISA assays, respectively. Values were mean ± SD from three independent experiments. ^*^ *P* < 0.05, ^**^ *P* < 0.01, ns not significant, vs BMSC (control) + bFGF. ^##^ *P* < 0.01 vs BMSC (control) + bFGF-PLGA and BMSC (anti-miR-29) + bFGF

We next investigated the effect of miR-29a-3p inhibition, when combined with bFGF-PLGA NPs, on the differentiation of BMSCs. Four different in vitro cultures were established: (1) BMSC (control) + bFGF, control BMSCs with 20 ng/ml free bFGF; (2) BMSC (control) + bFGF-PLGA, control BMSCs with 20 ng/ml equivalent bFGF-loaded PLGA NPs; (3) BMSC (anti-miR-29) + bFGF, miR-29a-3p inhibited BMSCs with 20 ng/ml free bFGF; (4) BMSC (anti-miR-29) + bFGF-PLGA, miR-29a-3p-inhibited BMSCs with 20 ng/ml equivalent bFGF-loaded PLGA NPs. All four groups of BMSC cultures were grown in proliferating media for 7 days, after which they were incubated in differentiating media for another 7 days. Cells were then collected to determine the elastin intracellular expression by RT-PCR and Western blot analyses, and their supernatant media were also collected to measure the elastin secretion using ELISA assay. We found significantly higher expressions of elastin mRNA and protein in both groups of miR-29a-3p-inhibited BMSCs than control BMSCs, regardless of free bFGF or bFGF-loaded PLGA (Fig. 5a and b). Interestingly, between the two miR-29a-3p-inhibited BMSC cultures, addition of bFGF-loaded PLGA NPs further upregulated elastin expression than free bFGF. As expected, elastin secretion into the media was also consistently higher from both miR-29a-3p-inhibited BMSC cultures, with bFGF-loaded PLGA NPs exhibiting higher extent than free bFGF (Fig. 5c). These results indicated that inhibiting miR-29a-3p in the BMSCs not only upregulated intracellular expression of elastin, but also stimulated its extracellular secretion.

### miR-29a-3p inhibition synergized with bFGF-PLGA in improving urodynamic tests in rat PFD model

We next established a rat PFD model to test the effect of miR-29a-3p-inhibited BMSC transplantation on PFD symptoms in vivo. Rats were assigned into six experimental groups (n = 12 each): (1) control, sham-operated rats to serve as healthy control; (2) PFD, vaginal distention was performed on the rats to induce PFD symptoms, and saline was injected 2 weeks after the operation; (3) BMSC (control) + bFGF, PFD rats were injected with control BMSCs and free bFGF 2 weeks after the operation; (4) BMSC (control) + PLGA-bFGF, PFD rats were injected with control BMSCs and bFGF-loaded PLGA 14 days after the operation; (5) BMSC (anti-miR-29) + bFGF, PFD rats were injected with miR-29a-3p-inhibited BMSCs and free bFGF 2 weeks after the operation; (6) BMSC (anti-miR-29) + PLGA-bFGF, PFD rats were injected with miR-29a-3p-inhibited BMSCs and bFGF-loaded PLGA 2 weeks after the operation. Subsequently, conscious cytometry (CMG) and leak point pressure (LPP) tests were conducted in all six groups of rats 7 days after injection.

**Fig. 6:**
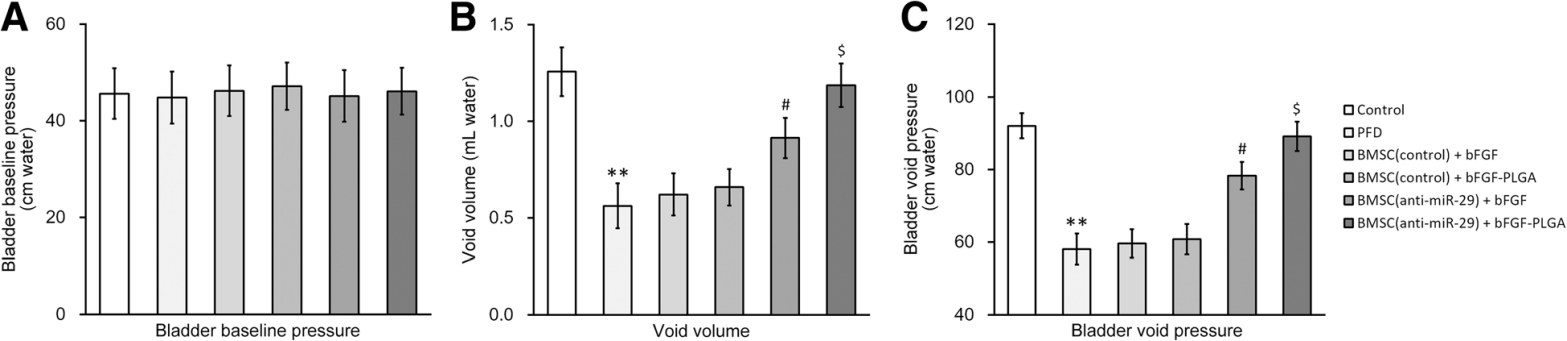
miR-29a-3p inhibition synergized with bFGF-PLGA in improving conscious cystometry in PFD rats after BMSC transplant. Rat PFD model was established and housed for 14 days, after which the rats were transplanted with BMSCs and housed for another 7 days. Conscious cytometry evaluations, including bladder baseline pressure (**a**), void volume (**b**), and bladder void pressure (**c**) were then performed on all rats in each experimental group. Values were mean ± SD (n = 12). ^**^ *P* < 0.01 vs control. ^#^ *P* < 0.05 vs PFD, BMSC (control) + bFGF and BMSC (control) + bFGF-PLGA. ^$^ *P* < 0.05 vs PFD, BMSC (control) + bFGF, BMSC (control) + bFGF-PLGA and BMSC (anti-miR-29) + bFGF

First, we observed similar baseline bladder pressure levels among all six groups of rats in CMG tests (Fig. 6a). In the PFD group rats, the void volume and bladder void pressure were greatly reduced compared to the sham-operated control rats (Fig. 6b and c, first two columns), indicating the establishment of the PFD model. Next, injections of control BMSCs with either free bFGF or bFGF-loaded PLGA NPs failed to restore void volume and bladder void pressure (Fig. 6b and c, third and fourth columns). Importantly in rats receiving injections of miR-29a-3p-inhibited BMSCs, the decreased void volume and bladder void pressure were both rescued (Fig. 6b and c, fifth and sixth columns), with defects in BMSC (anti-miR-29) + bFGF-PLGA group rats almost completely restored to similar levels of the sham-operated control rats (Fig. 6b and c, sixth column).

**Fig. 7:**
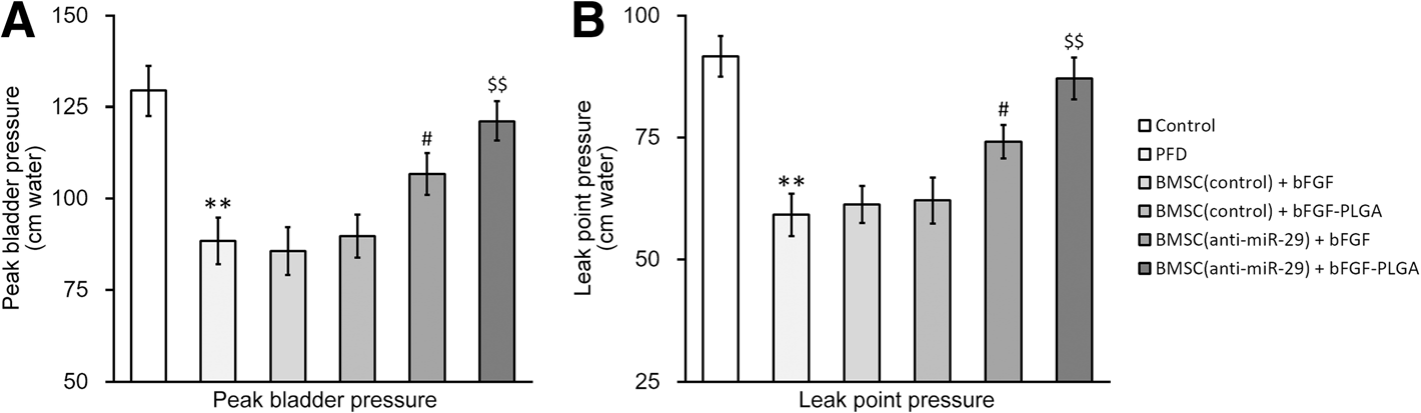
miR-29a-3p inhibition synergized with bFGF-PLGA in improving leak point pressure in PFD rats after BMSC transplant. PFD rat model was established and housed for 14 days, after which the rats were transplanted with BMSCs and housed for another 7 days. Evaluations of peak bladder pressure (**a**) and leak point pressure (**b**) were then performed on all rats in each experimental groups. Values were mean ± SD (n = 12). ^*^ *P* < 0.05 vs control. ^#^ *P* < 0.05 vs PFD, BMSC (control) + bFGF and BMSC (control) + bFGF-PLGA. ^$$^ *P* < 0.01 vs PFD, BMSC (control) + bFGF, BMSC (control) + bFGF-PLGA and BMSC (anti-miR-29) + bFGF

Results from the LPP test displayed a very similar pattern. In the PFD rat group, both peak bladder pressure and leak point pressure were significantly lower than the sham operated control rats (Fig. 7a and b, first two columns). As expected, injection of control BMSCs with either free bFGF or bFGF-loaded PLGA NPs exhibited almost no effect on peak bladder and leak point pressures (Fig. 7a and b, third and fourth columns). In contrast, injection of miR-29a-3p inhibited BMSCs restored void volume and bladder void pressure (Fig. 7a and b, fifth and sixth columns). In addition, co-injection of miR-29a-3p inhibited BMSCs together with bFGF-loaded PLGA fully reversed the defective peak bladder and leak point pressures (Fig. 7a and b, sixth column).

Taken together, regardless of bFGF source, transplant of control BMSCs did not exhibit any alleviating effect on the PFD rats. However when injected together with the bFGF-loaded PLGA NPs, genetically engineered miR-29a-3p-inhibited BMSCs significantly improved results of urodynamic tests to comparable levels of the sham-operated control rats.

## Discussion

Elastin is the major component of the elastic fibers in the extra cellular matrix (ECM) of many types of connective tissues such as the ligaments and the aorta. It functions to facilitate the stretch and return of tissues to their original form in the absence of energy. Metabolism of elastin has been shown to be affected in PFD as a result of abnormal synthesis, interruption in homeostasis, as well as increased degradation [19–22]. Weakened connective tissues in the pelvis are thought to contribute to pathophysiological development of PFD. Since elastin is an important component of connective tissues in the vagina and pelvic floor, a growing body of studies has begun to implicate elastin in the pathogenesis of PFD, particularly in SUI and POP, which are two major conditions frequently linked to childbirth injuries. In the process of delivery, vaginal distention can induce degradation of the elastin fibers, whose normal organization is then disrupted, causing malfunctions that ultimately increase stiffness of connective tissues. Previous study using a defective elastin mouse model has demonstrated that synthesis of elastic fibers is a significant process for recovery after childbirth, and compromised elastin synthesis could result in prolapse [23].

In our previous proof-of-concept study, we pioneered in a multidisciplinary therapy, involving an elastin gene modification, stem cells, and nanoparticle technology, and demonstrated its efficacy in the repair and regeneration of pelvic floor tissues in PFD rats [12]. In this current work, we have further expanded the scope of the therapeutic concept, to explore other potential regulatory factors, such as microRNAs (miRNAs or miRs). MicroRNAs (miRNAs or miRs) are a class of 21-25 nucleotide long, small noncoding RNA molecules, which recognize specific complementary sequences predominantly found in the 3′-UTR of target mRNAs, to either repress translation or degrade these mRNAs [24, 25]. In our current study, we found that in BMSCs, miR-29a-3p was able to directly target and suppress the expression of elastin. Downregulation of elastin by miR-29 family miRNAs has been widely reported. For instance, during postnatal aortic development, miR-29 was found to be differentially expressed to downregulate elastin [14]. In cells haploinsufficient for elastin and in bioengineered vessels, inhibition of miR-29 could increase elastin expression levels [15]. Recently in a study performed in vascular smooth muscle cells, miR-29-mediated elastin downregulation was found to contribute to osteoblastic differentiation induced by inorganic phosphorus [16]. Results from the above studies consistently point to an important role of miR-29, miR-29a-3p in particular, in our current work, in regulating the expression of elastin in the mammalian system, including BMSCs.

The potential of BMSCs in soft tissue repair and reconstruction has been widely reported [26, 27]. For instance, transplant of BMSCs promoted growth of new tissues as well as deposited collagen in an in vivo wound-healing animal model [28]. Particularly in the treatment of PFD, maintaining the right amount of elastic fibers in the connective tissues is extremely important to repair damage in the pelvic floor and restore its support functions. However, direct collagen deposit could induce formation of dense connective and scar tissue, and a potential solution is elastin gene-engineered cell therapy [29], in which elastin overexpression in BMSCs was shown to restore elasticity in the dilated cardiac tissues and subsequently cardiac functions. In line with the above study by Li et al., as well as our previously work [12], inhibition of miR-29a-3p increased expression of elastin in BMSCs, which after being injected into PFD rats restored the function of tissues damaged by virginal distention.

Last but not least, in a very recent study [30], a comparison of miRNA expression profile between the dermis tissues of the young and elderly revealed that, several miRNAs, including miR-29, were upregulated in aged dermis. Given the important regulatory role of miR-29a-3p in suppressing elastin expression, it will be of particular interest to our current work to investigate if miR-29a-3p is also differentially expressed in human PFD patients as well.

## Conclusions

In conclusion, we have demonstrated the role of miR-29a-3p as an important negative regulating factor of endogenous elastin expression in BMSCs. Inhibition of miR-29a-3p in BMSCs resulted in markedly higher expression and secretion of elastin, which consequently promoted the therapeutic potential of the BMSCs following injection into PFD rats. Our multidisciplinary study, combining microRNA biology, genetically engineered BMSCs, and nanoparticle technology, provides not only an excellent combinational therapy for repairing connective tissues, but also an in vivo experimental platform to search for other potential therapeutic factors or targets of PFD.
